# Functional Analysis of the *Acinetobacter baumannii* XerC and XerD Site-Specific Recombinases: Potential Role in Dissemination of Resistance Genes

**DOI:** 10.3390/antibiotics9070405

**Published:** 2020-07-13

**Authors:** David L. Lin, German M. Traglia, Rachel Baker, David J. Sherratt, Maria Soledad Ramirez, Marcelo E. Tolmasky

**Affiliations:** 1 Center for Applied Biotechnology Studies, Department of Biological Science, California State University Fullerton, Fullerton, CA 92831, USA; davlin0916@gmail.com (D.L.L.); msramirez@Fullerton.edu (M.S.R.); 2 Department of Biochemistry, University of Oxford, Oxford OX1 3QU, UK; rachel.baker@bioch.ox.ac.uk (R.B.); david.sherratt@bioch.ox.ac.uk (D.J.S.); 3 Departamento de Desarrollo Biotecnológico, Instituto de Higiene, Facultad de Medicina, Universidad de la República (UDeLaR), Montevideo 11600, Uruguay; gertra13b@gmail.com

**Keywords:** site-specific recombination, carbapenemase, ESKAPE, *Acinetobacter*, plasmid, Xer, dif, pdif, Re27, gene transfer, gene dissemination, horizontal dissemination, horizontal transfer

## Abstract

Modules composed of a resistance gene flanked by Xer site-specific recombination sites, the vast majority of which were found in *Acinetobacter baumannii*, are thought to behave as elements that facilitate horizontal dissemination. The *A. baumannii* *xerC* and *xerD* genes were cloned, and the recombinant clones used to complement the cognate *Escherichia coli* mutants. The complemented strains supported the resolution of plasmid dimers, and, as is the case with *E. coli* and *Klebsiella pneumoniae* plasmids, the activity was enhanced when the cells were grown in a low osmolarity growth medium. Binding experiments showed that the partially purified *A. baumannii* XerC and XerD proteins (XerC_Ab_ and XerD_Ab_) bound synthetic Xer site-specific recombination sites, some of them with a nucleotide sequence deduced from existing *A. baumannii* plasmids. Incubation with suicide substrates resulted in the covalent attachment of DNA to a recombinase, probably XerC_Ab_, indicating that the first step in the recombination reaction took place. The results described show that XerC_Ab_ and XerD_Ab_ are functional proteins and support the hypothesis that they participate in horizontal dissemination of resistant genes among bacteria.

## 1. Introduction

Site-specific recombination mediated by the tyrosine recombinases XerC and XerD (XerCD SSR) participates in a wide array of genetic processes in bacteria. After activation by FtsK, XerC and XerD catalyze the resolution of dimeric chromosomes formed by homologous recombination as a consequence of repaired, broken, or stalled replication forks [1,2,3]. XerCD SSR is critical for stabilization of numerous plasmids by resolving multimers that otherwise would lead to segregational instability [4,5]. In this case, the recombination reaction requires the presence of architectural proteins, like PepA and ArgR or ArcA, to form of a synaptic complex that acts as a topological filter, permitting the resolution but not formation of multimers [6]. At least for some *Escherichia coli* and *Klebsiella pneumoniae* plasmids, XerCD SSR activity levels depend on the osmolarity of the environment that produce modifications in the supercoiling density [7,8,9,10]. Many genetic elements take advantage of XerCD SSR to integrate into the bacterial chromosome. These elements, the vast majority of them phages, are known as IMEX (Integrative Mobile Elements that integrate through XerCD SSR), of which three different classes have been described to date [11,12,13,14,15]. XerCD SSR also participates in plasmid evolution as the mechanism of resolution of cointegrates formed between different plasmids by recombination at the *oriT* sites [16,17,18]. More recently, resistance genes residing in *Acinetobacter baumannii* plasmids were found flanked by XerC- and XerD-like binding sites (XerC/D binding sites), also referred to as Re27 or p*dif* [19,20]. The common presence of these Xer modules (XerC/D binding sites–resistance gene–XerC/D binding sites) led to the proposal that these elements play an important role in the horizontal dissemination of resistance genes [19,21,22,23,24,25,26]. The interest in the Xer modules was enhanced because many of them include carbapenemase genes like *bla*_OXA24/40_-like, which confer *A. baumannii*, a member of the ESKAPE group of bacteria [27], the ability to resist some of the last-line antibiotics [21,22,23,24,25,28]. Xer modules have also been recently found in bacteria other than *Acinetobacter* and including genes other than *bla*_OXA24/40_-like. These findings suggested that XerCD SSR may play a more general role in horizontal dissemination of different gene classes among multiple bacteria [19,23,26]. Among the genes found within these modules are *bla*_OXA-58_; the *bla*_OXA-143_-like *bla*_OXA-253_; the tetracycline resistance gene *tet39*; the macrolide resistance genes *msrE* and *mphE*; the chromium resistance genes *chrA* and *chrB*; the organic hydroperoxide resistance genes *ohr/ohrR*; the transport-related genes, such as *sulP* and *kup*; the tellurium resistance *terC*; and toxin-antitoxin (*add*) genes [19,23,26,29,30,31]. Some of them, like *ohr/ohrR*, *chrA*, *chrB*, *sulP*, *kup*, and *add*, were found in different *Acinetobacter* species [30], and *bla*_OXA-58_ was found in *Proteus mirabilis* [26,32].

Most of the evidence of the involvement of XerCD SSR in gene mobilization in *Acinetobacter* was inferred from analysis of nucleotide sequences. The only indirect experimental evidence of Xer recombination in *Acinetobacter* was obtained after two *A. baumannii* plasmids were introduced in *A. nosocomialis*, where they formed a cointegrate by recombination at the XerC and XerD binding sites [33]. In this work we report the cloning and functional characterization of the *A. baumannii* XerC and XerD (XerC_Ab_ and XerD_Ab_).

## 2. Results

### 2.1. Cloning and Complementation Analysis of A. baumannii Xer Recombinases

**Figure 1:**
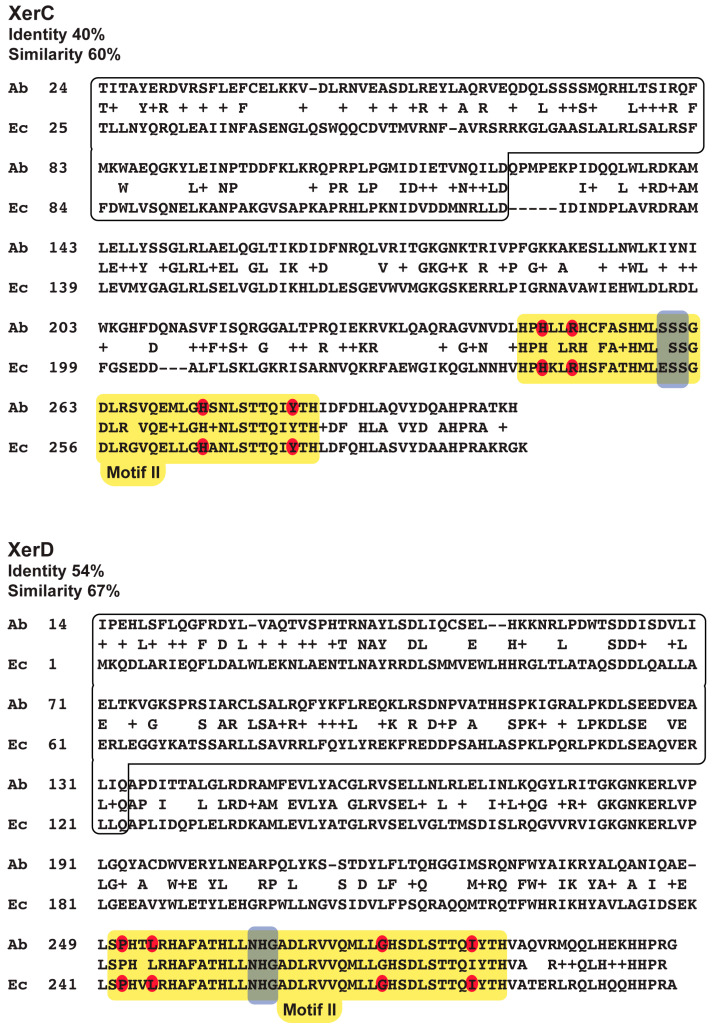
Alignment of the amino acid sequences of the *A. baumannii* and *E. coli* XerC and XerD. Catalytic, conserved amino acids are highlighted in red. The motif II is indicated by a solid yellow box. The tripeptides that act as acceptor in the donor–acceptor interaction with the C-terminal end of the partner protein are highlighted inside a solid gray box. The N-terminal region is boxed. Amino acid sequences are from accession numbers NP_418256.1 (*E. coli* MG1655 XerC), NP_417370.1 (*E. coli* MG1655 XerD), [43], VCCO00000000 (*A. baumannii* A118 XerC and XerD) [44].

XerC and XerD proteins are characterized by possessing two domains, the C- and N-terminal, attached by a linker [34,35]. The C-terminal domains recognize the outer nucleotide sequences of their binding sites, including the catalytic amino acids, and participate in recombinase–recombinase interactions that coordinate their activity (Figure 1) [34,36,37]. The main N-terminal domains’ role is the recognition and binding to the inner segment of the binding sites (boxed in Figure 1) [38]. XerC and XerD control their catalytic activity through interactions where the C-terminal portion of one of the proteins (donor) contacts the other (acceptor) in a donor–acceptor fashion. Amino acids at the donor C-terminal region interact with a stretch of three amino acids in the acceptor (boxed in gray in Figure 1), producing a conformational change that activates the latter [36]. This interaction produces a modification in the folding of the recipient protein that positions the catalytic amino acid residues for recombination to proceed [36]. The recipient amino acids in the *E. coli* XerC and XerD proteins are ESS and NHG, respectively (Figure 1) [34,36]. The XerC and XerD C-terminal regions that include the tripeptide and four catalytic amino acids are known as motif II (boxed in yellow in Figure 1) [35,36]. Recombination at some sites like the chromosome’s *dif* occurs through sequential activation of the recombinases where one catalyzes the exchange of the first pair of strands to form a Holliday junction, and then the activation of the second completes the reaction [1]. In other cases, like the ColE1 plasmid *cer* [39] or the pJHCMW1 plasmid *mwr* [40], XerD is needed to activate XerC, which catalyzes the formation of the Holliday junction, which is then processed independently of XerD, most probably by DNA replication [9,10,41,42]. 

The amino acid sequences of all XerC_Ab_ and XerD_Ab_ proteins deposited in GenBank were compared and they share high identity (Supplementary Material, Figure S1). Although the overall XerC and XerD amino acid sequences from *E. coli* and *A. baumannii* share low identity (40% and 54% identity, respectively), there is a higher degree of identity and similarity at their motif II regions. Figure 1 shows a comparison of the *E. coli* and *A. baumannii* XerC and XerD amino acid sequences. The XerD motif II amino acid sequences are nearly identical, and there is some divergence in the XerC amino acid sequences of the same region, including one of the amino acids in the tripeptide that interacts with XerD. Nonetheless, the amino acid sequences are close enough to expect that the heterologous proteins can interact. Then, we hypothesized that at least partial activity will be observed in complementation experiments where the *A. baumannii* enzyme is introduced in the corresponding *E. coli* mutant. A similar approach was used before to characterize XerCD SSR in *K. pneumoniae* [7].

**Figure 2:**
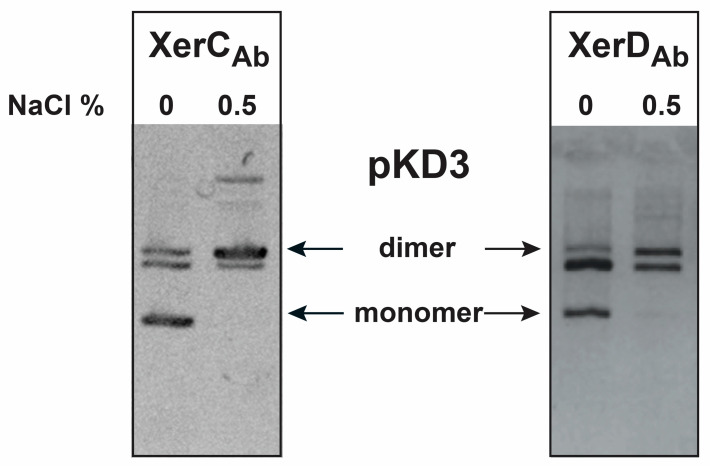
Resolution of plasmid dimers. Dimers of the plasmid pKD3 were introduced by transformation into *E. coli* DS981XerC_Ab_ or *E. coli* DS99028XerD_Ab_. The cells were cultured overnight in a low or high osmolarity medium in the presence of 100 μg/mL of ampicillin. Plasmid DNA was isolated and subjected to agarose gel electrophoresis. The bands below the dimer correspond to the complementing plasmid. The position of migration of the dimers and monomers are indicated to the sides.

The *xerC*_Ab_ and *xerD*_Ab_ genes were cloned and the recombinant clones, pMSR1 and pMSR2, were transferred to the corresponding *E. coli* mutants to assess their recombination activity in the plasmid dimer resolution assays. *E. coli* DS981XerC_Ab_ and DS9028XerD_Ab_ were transformed with dimers of the plasmid pKD3 and cultured overnight in medium containing 0.5% (high osmolarity) or no NaCl added (low osmolarity) before extracting the plasmid DNA. Analysis of the plasmid content from both strains showed similar results (Figure 2). While resolution was almost undetectable in cells growing in a high osmolarity medium, most of the plasmid DNA was found in its monomeric form in cells growing in a low osmolarity medium (Figure 2). These results indicated that both XerC_Ab_ and XerD_Ab_ were active in *E. coli*. The differences in levels of resolution when the cells were cultured in a high or low osmolarity medium were not surprising. Resolution of plasmid dimers at many XerCD target sites is dependent on the osmolarity of the growth medium [8,45]. Modifications in the osmolarity of the environment induce changes in the topology of the plasmid molecules making them more suitable substrates [10]. 

### 2.2. Binding of A. baumannii Xer Recombinases to XerC/D Binding Sites

**Figure 3:**
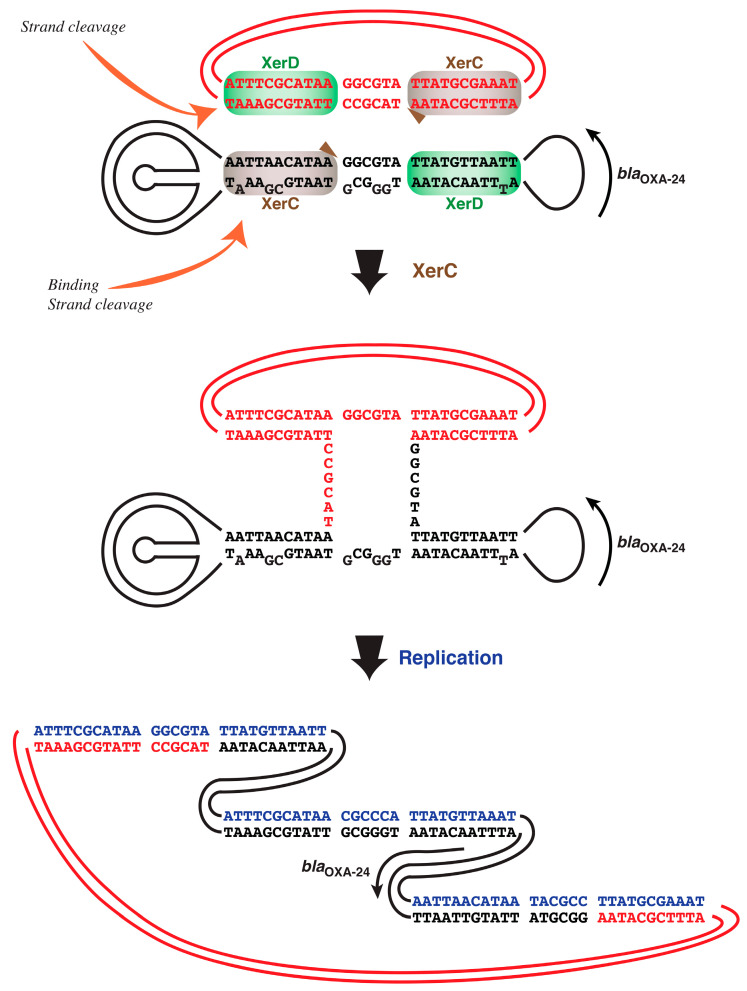
Hypothetical generation of the XerC/D binding sites found in the plasmid pMMCU1. The diagram shows a hypothetical recombination/replication process between two DNA molecules that produced the structure found in pMMCU1. The molecule represented in black includes blaOXA-24 flanked by XerC/D binding sites in opposite orientations. As a consequence, both strands can fold into imperfect hairpin structures that create XerC/D binding sites. The red molecule includes a XerC/D binding site that recombines with that formed by the black molecule hairpin. Blue nucleotides are those in the newly replicated strand. The nucleotide sequences and arrangement of the plasmid at the bottom are those of the plasmid pMMCU1 (accession number GQ342610) [21]. Orange arrows show the experiments performed using the oligonucleotides pointed.

**Figure 4:**
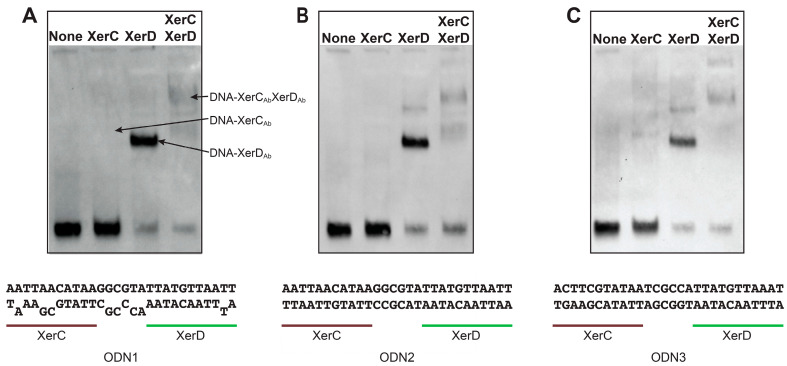
XerC_Ab_ and XerD_Ab_ binding to recombination sites. Labeled oligodeoxynucleotides were incubated in the absence or presence of the proteins indicated at the top. The products were separated by electrophoresis in an 8% polyacrylamide gel and treated as described in Materials and Methods. The nucleotide sequences of the potential Xer recombination sites tested are shown below the gels. (**A**) XerC and XerD binding sites identical to *dif* and numerous *A. baumannii* plasmids. (**B**,**C**) Matched and mismatched sites from the progenitor black molecule, respectively (see Figure 3).

*Acinetobacter* plasmids often include clinically relevant resistance genes, such as *bla*_OXA-24_ or its close relative *bla*_OXA-72_, flanked by XerC/D binding sites. Although originally these were structures uniquely found in *A. baumannii*, further studies showed that other genes were part of similar structures found in *Acinetobacter* or other genera [19,20,21,22,23,24,25,26,28,29,30,31]. These findings led to propose that Xer modules (XerC/D binding sites-resistance gene-XerC/D binding sites) are elements that facilitate the horizontal dissemination of resistance genes by XerCD SSR. However, this hypothesis is mostly based on nucleotide sequencing analyses. The first step in XerCD SSR is the cooperative attachment of XerC and XerD to the putative XerC/D binding sites. To test if XerC_Ab_ and XerD_Ab_ bind to the appropriate sites, oligodeoxynucleotides were designed taking into consideration the XerC/D binding sites identified in the *A. baumannii* plasmid pMMCU1 [21,46]. The arrangement XerC/D binding site–XerC/D binding site–*bla*_OXA-24_–XerC/D binding site was used to identify the potential nucleotide sequence of the XerC/D binding sites of two hypothetical DNA molecules that could have originated the arrangement found in pMMCU1 after XerCD SSR. Figure 3 shows the nucleotide sequences of the hypothetical original XerC/D binding sites and the Xer recombination/replication events that could have taken place. This recombination pathway has been proposed for reactions mediated by Xer as well as for other recombination systems such as the integration of gene cassettes catalyzed by IntI1 [12,13,47,48]. In this pathway, one of the recombination sites is generated by formation of a hairpin between two XerC/D binding sites located in opposite orientations (see Figure 3). In the hypothetical molecule shown in black in Figure 3, the sequences that form the hairpin are not perfectly complementary. Therefore, to test binding of XerC_Ab_ and XerD_Ab_, two oligonucleotides were synthesized, one conserving the predicted mismatches (ODN1) and another one fully complementary (ODN2) (Table 1; see also Figure 3 and Figure 4). Another tested oligodeoxynucleotide, ODN3 (Table 1), has the potential XerC and XerD binding regions found in at least 100 *A. baumannii* plasmids (as determined by BLASTN) and in the *A. baumannii dif* locus [49].

Figure 4 (Panels A, B, and C) shows that both XerC_Ab_ and XerD_Ab_ cooperatively bound the XerC/D binding sites tested. XerC_Ab_ showed a weak binding capability when tested in the absence of XerD_Ab_, a property that is usually observed with Xer recombinases from other bacteria [50].

### 2.3. A. baumannii Xer Recombinases-Mediated Strand Exchange

**Figure 5:**
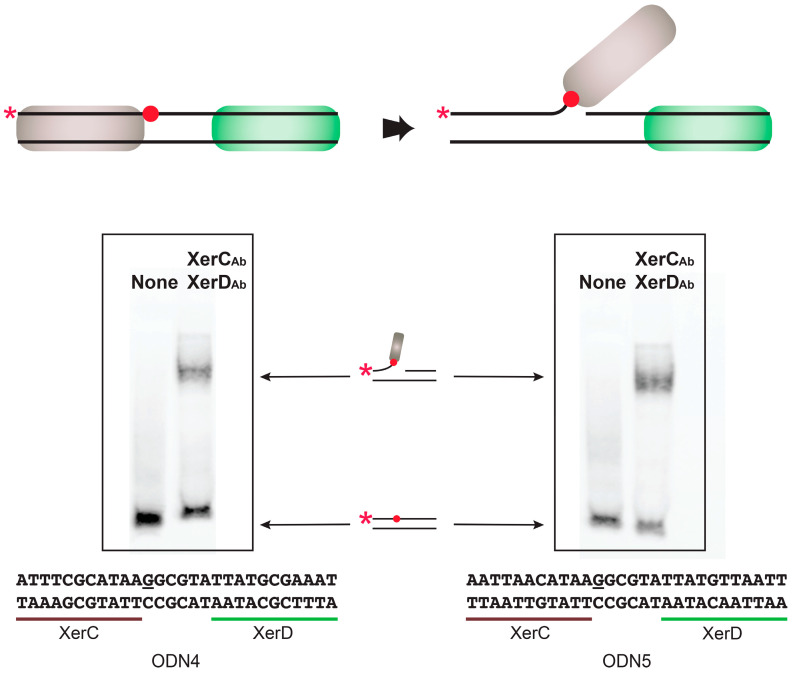
In vitro cleavage of the Xer recombination sites. The substrate double-stranded oligonucleotides include a phosphorothioate analog (underlined, red dot) to trap the DNA-Xer product formed after digestion and covalent attachment to the Y residue of the recombinase (top). Reactions were carried out at 37 °C for 1 h in the presence or absence of XerC_Ab_ (brown) and XerD_Ab_ (green). Samples were heated at 95 °C for 5 min and subjected to 1% sodium dodecyl sulfate–8% polyacrylamide gel electrophoresis. The asterisk represents the 5′-end biotinylation. The bands were visualized as described in Materials and Methods.

After the binding of XerC and XerD to their respective binding sites, the following step in the recombination reaction is the strand exchange, which requires that the DNA is cleaved and covalently bound to a tyrosine residue in the recombinase (Figure 5). The two putative XerC/D binding sites in the pMMCU1 predecessor molecules shown in Figure 3 were utilized in suicide substrate cleavage assays. The top strand, the one digested by XerC, was synthesized with a phosphorothioate residue at the point of cleavage. Therefore, the Xer recombinase forms an irreversible covalent bond with the 3′-end of the top oligodeoxynucleotide containing the sulfhydryl group from the 5′-end of the nick. Incubation of both substrates with XerC_Ab_ and XerD_Ab_ followed by heat denaturation produced a high molecular weight band indicating that a Xer recombinase, presumably XerC, is covalently bound to the substrate (Figure 5). These results demonstrated that the *A. baumannii* XerC and XerD proteins are capable of mediating the recombination reaction necessary to facilitate dissemination of genes flanked by the XerC/D binding sites.

## 3. Discussion

XerCD SSR is now known to play numerous biological roles, such as ensuring the survival of a substantial percentage of individuals in a bacterial population that suffered dimerization of the chromosome, unlinking the chromosome catenanes [51,52], preventing plasmid segregational instability that would result if dimer formation remains unresolved [4,5], catalyzing integration of IMEXs into the bacterial chromosome [12,13,14], and participating in plasmid evolution using diverse mechanisms [16,17,18]. The analysis of nucleotide sequences of numerous plasmids and genomes, especially in *A. baumannii*, led to the proposal that genes flanked by the Xer site-specific recombination sites, mostly in opposite orientations, conform elements that mediate dissemination of the genes [19,20,21,22,23,24,25,26,28,29,30,31]. The role of the XerCD SSR in disseminating resistance genes, such as those coding for carbapenemases—enzymes that elevated *A. baumannii* to the category of one of the most important threats to human health [28,53,54]—underscore the relevance of understanding XerCD SSR in this bacterium.

The functionality of XerC_Ab_ and XerD_Ab_ was first demonstrated by cloning of the cognate genes and using the recombinant clones to complement the *E. coli* mutants. The divergence in the *A. baumannii* and *E. coli* amino acid sequences was expected to result in lower than ideal levels of resolution. On the other hand, motif II showed high conservation, which permitted the interaction between the heterologous XerC and XerD to activate the recombination reaction. Dimer resolution in the complemented *E. coli* strains was minimal when the cells were cultured in a high osmolarity growth medium, but it was substantially higher in a low osmolarity medium. These results showed that XerC_Ab_ and XerD_Ab_ are functionally proficient in stabilizing plasmids by dimer or multimer resolution. As is the case for numerous plasmids [8,45], the recombination reaction was also strongly dependent on the concentration of osmolites in the growth medium. The resolution of dimers by XerCD SSR requires that the target site includes accessory sequences in addition to the core recombination site [6]. Instead, the target sites that flank the resistance genes in the Xer modules lack accessory sequences. To initiate a study of the recombination at these sites, XerC_Ab_ and XerD_Ab_ were partially purified and tested in vitro using as substrates the common Xer recombination sites or sites that were designed after theoretical reverse engineering of the arrangement found in pMMCU1 [21]. These potential Xer target sites could have originated pMMCU1 by Xer recombination (see Figure 3). All the tested sites showed cooperative binding by XerC_Ab_ and XerD_Ab_. They also showed low binding when only XerC_Ab_ was present, but the binding was more efficient with XerD_Ab_ or both proteins. This result is similar to those observed with the *E. coli* XerC and XerD in binding experiments using numerous target sites [8,9,50]. Cleavage experiments using suicide substrates showed that the proteins are active beyond binding. The cleavage and covalent bond to, most probably, XerC, proved that the predicted sites that originated the pMMCU1 Xer site-specific recombination sites arrangement are suitable substrates of the *A. baumannii* recombinases. XerCD SSR can occur through two-step strand exchanges or a pair of strand exchange followed by replication; in this latter case the original two molecules become cointegrated (Figure 3). In both cases, a resistance gene becomes part of a molecule with a new replicon, which could replicate in a different set of bacterial genera. As a consequence, successive rounds of recombination have the potential to greatly expand the range of bacteria that become resistant.

## 4. Materials and Methods 

### 4.1. Bacterial Strains and Plasmids

The plasmids and strains used in this work are described in Table 2. *E. coli* DS941 possesses all the genes involved in Xer recombination. It was originally used to generate *xerC* and *xerD* mutant derivatives [55]. *E. coli* DS981 (DS941 *xerC2*::*aph*) (KAN^r^) [56] and DS9028 (DS941 *xerD3*::*fol*) (TMP^r^) [57] were used in complementation experiments. *E. coli* JC8679 (hyperrecombinogenic) [39] was used to generate plasmid dimers. *A. baumannii* A118 is a clinical isolate that was used as source of *xerC*_Ab_ and *xerD*_Ab_ genes [58,59]. A subscript indicates if the gene or protein is from *A. baumannii* or *E. coli*, e.g., *xerC*_Ab_ or *xerC*_Ec_. Plasmids pUC18 [60], pCR2.1 (Life Technologies Co.), and pACYC184 [61] were used as vectors in cloning experiments. Plasmid pKD3 is pUC18 with an insertion of a DNA fragment, including the Xer recombination site *mwr*_T_ [8]. 

### 4.2. General DNA Procedures

Bacteria were cultured in Lennox L broth (1% tryptone, 0.5% yeast extract, 0.5% NaCl), and 2% agar was added in the case of solid medium. Transformations were carried out as described by Cohen et al. [62]. Plasmid DNA preparations and DNA gel extractions were performed with the QIAprep Spin miniprep kit and QIAquick gel extraction kit, respectively (QIAGEN). Restriction endonuclease and ligase treatments were performed as recommended by the suppliers. DNA fragments containing the *xerC*_Ab_ or *xerD*_Ab_ genes were generated by PCR amplification with the QIAGEN Taq master mix using as a template the genomic DNA from *A. baumannii* A118. Amplicons were inserted in pCR2.1 and then subcloned into the *Eco*RI site of pACYC184. Both *xerC*_Ab_ or *xerD*_Ab_ were further subcloned into pBAD102 with a C-terminal 6x histidine tag for overexpression and purification. The inserts of all recombinant plasmids were sequenced to ensure accuracy. Nucleotide sequencing was performed at the DNA Sequencing Facility, Department of Biochemistry, University of Oxford.

### 4.3. Protein Purification 

C-terminally tagged XerC_Ab_ and XerD_Ab_ with 6x histidine were affinity purified using TALON metal affinity resin as previously described [41]. Briefly, *E. coli* DS9040 (pBAD102*xerC*_Ab_) or *E. coli* DS9040 (pBAD102*xerD*_Ab_) were cultured overnight at 37 °C with shaking. Each culture was then diluted 1:100 and shaken at 200 rpm at 37 °C for 3.5 h. At this moment, protein expression was induced by addition of 0.1% arabinose and incubation at 30 °C. After 4 h, the cells were collected by centrifugation at 5000 rpm for 20 min and resuspended in a buffer containing 50 mM Tris 7.5, 1 M NaCl, and 10% glycerol with protease inhibitor cocktail (Sigma). The cells were lysed using a French Press and the lysate was subjected to centrifugation at 19,000 rpm for 30 min at 4 °C. The supernatant containing the protein of interest was mixed with TALON metal affinity resin and incubated for 1 h. The resin was washed with a buffer containing 50 mM Tris-HCl (pH 7.5), 500 mM NaCl, 10% glycerol, and 10 mM imidazole. The proteins were eluted by a gravity column into 8 fractions with a buffer containing 50 mM Tris-HCl (pH 7.5), 500 mM NaCl, 10% glycerol, and 200 mM imidazole. Proteins were analyzed using sodium dodecyl sulfate–15% polyacrylamide gel electrophoresis stained with Coomassie blue to identify the fractions containing XerC_Ab_ and XerD_Ab_ (Supplementary Material, Figure S2). The selected fractions were pooled, dialyzed with 10 mM Tris-HCl (pH 7.5) using Zeba desalting columns (ThermoFisher Scientific), and concentrated to approximately 150 μg/mL using Pierce protein concentrator PES columns (ThermoFisher Scientific) according to the manufacturer’s recommendations. 

### 4.4. In Vivo Resolution Assay

In vivo resolution assays were carried out basically as described by Pham et al. [8]. Plasmid dimers, generated using *E. coli* JC8679 as described previously [8], were introduced by transformation into the indicated strains. The transformant strains were cultured overnight at 37 °C in Lennox L broth (high osmolarity) or medium containing the same concentrations of tryptone and yeast extract as the Lennox L broth but without the addition of NaCl (low osmolarity). After overnight growth, plasmid DNA was purified and analyzed by electrophoresis in a 0.7% agarose gel. 

### 4.5. DNA-Binding Assay

Binding of XerC_Ab_ and XerD_Ab_ to the potential recombination sites were carried out as described before [9,63]. An oligodeoxynucleotide was 5′-end biotinylated using the 5′-EndTag DNA labeling kit. Equal volumes of equimolar solutions (in NEB Cutsmart buffer) of the labeled compound and the complementary oligodeoxynucleotide were mixed, heated at 95 °C for 5 min, let slowly cooldown to room temperature, and placed on ice, to generate a labeled, double-stranded XerC/D recombination site. An aliquot containing 40 fmols of the DNA substrate was mixed with a reaction buffer (10 mM Tris-HCl (pH 7.5), 50 mM KCl, 1 mM DTT, 25 ng/μL poly dI/dC, 2.5% glycerol, 10 mM EDTA, and 0.05% NP-40). The indicated protein (XerC, XerD, or both) was added to the reaction mix at a final concentration of 150 ng/μL. The reaction was incubated at 37 °C for 1 h. Samples were analyzed using 8% polyacrylamide gel electrophoresis at 100 V. Resolved DNA and protein complexes were transferred to a nylon membrane using the iBlot system (ThermoFisher Scientific), following the recommendation of the supplier. After the transfer, blots were cross-linked under UV light in a Stratalinker 1200 for 120 s. The blots were visualized using the Chemiluminescent Nucleic Acid Detection Module Kit (ThermoFisher Scientific). Bands were visualized by ChemiDoc XRS (BioRad).

### 4.6. In Vitro Xer-Mediated DNA Cleavage

Digestion by and covalent binding to XerC_Ab_ was carried out using a labeled, double-stranded oligodeoxynucleotide, in which the top strand includes a phosphorothioate analog residue at the point of digestion and a 5′ biotin modification. When this substrate is nicked, the Xer recombinase forms a covalent bond between the 3′-end of the oligodeoxynucleotide containing the sulfhydryl group from the 5′-end of the nick and the tyrosine for the recombinase. The 5′-SH group is a poor nucleophile for religation, making the reaction irreversible. In these conditions, there is an accumulation of the DNA-recombinase covalent product [64]. An aliquot containing 0.8 pmols of annealed dsDNA was mixed with a buffer containing 20 mM Tris-HCl (pH 7.5), 50 mM NaCl, 0.1 mM EDTA, 2.5% glycerol, and 50 ng/μL bovine serum albumin. Aliquots containing XerC_Ab_ and XerD_Ab_ were added to the reaction to a final concentration of 150 ng/μL of each protein and incubated at 37 °C for 1 h. The reactions were terminated by adding a denaturing loading buffer containing an additional 2% sodium dodecyl sulfate and 5% β-mercaptoethanol. Samples were heated at 95 °C for 5 min and subjected to 1% sodium dodecyl sulfate–8% polyacrylamide gel electrophoresis. The biotinylated DNA and protein complexes were transferred to a nylon membrane and visualized as described above.

**Table 2:** Bacterial strains and plasmids used in this study.

Bacterial Strain or Plasmid	Relevant Characteristics, Genotype, or Phenotype ^a^	Source or Reference
***E. coli* strains**		
DS941	AB1157 *recF143 lacI*^q^ *lacZ*ΔM15	[65]
DS981	DS941 *xerC* (Kan^r^)	[56]
DS9028	DS941 *xerD* (Tmp^r^)	[57]
DS981XerC_Ab_	DS981 (pMSR1) (Kan^r^ Tet^r^)	This work
DS9028XerD_Ab_	DS9028 (pMSR2) (Tmp^r^ Tet^r^)	This work
DS9040	DS941 *xerC xerD* (Kan^r^ Gen^r^)	[34]
JC8679	DS945 *recBC sbcA* (hyperrecombinogenic)	[39]
***A. baumannii* strain**		
A118	Human clinical isolate	[58]
**Plasmids**		
pMSR1	*xerC*_Ab_ cloned into the pACYC184 *Eco*RI site (Tet^r^)	This work
pMSR2	*xerD*_Ab_ cloned into the pACYC184 *Eco*RI site (Tet^r^)	This work
pBAD102*xerC*_Ab_	*xerC*_Ab_ cloned into pBAD102 (Amp^r^)	This work
pBAD102*xerD*_Ab_	*xerD*_Ab_ cloned into pBAD102 (Amp^r^)	This work
pKD3	*Eco*RI-*Sac*I fragment containing the pJHCMW1 *mwr* site with substitution C to T at the ArgR binding site cloned in pUC18 (Amp^r^)	[8]
pUC18	Cloning vector (Amp^r^)	[60]
pCR2.1	Cloning vector (Amp^r^, Kan^r^)	ThermoFisher
pACYC184	Cloning vector, p15A replicon (Chl^r^ Tet^r^)	[61]

^a^ Amp, ampicillin; Chl, chloramphenicol; Gen, gentamicin; Kan, kanamycin; Tet, tetracycline; Tmp, trimethoprim.

## 5. Conclusions

This article describes the biological evidence of functional XerC and XerD recombinases in *A. baumannii.* The proteins were cloned, partially purified, and tested in vitro to show their ability to catalyze recombination between sites recognized in the literature as putative Xer site-specific recombination sites that are part of the antibiotic resistance gene-mobilization modules postulated in the literature. This article also shows that XerC_Ab_ and XerD_Ab_ can participate in plasmid stability by multimer resolution. 

## Figures and Tables

**Table 1:** Oligonucleotides used in the binding and cleavage assays.

Name	Sequence
ODN1	A(A/A)TT(A/G)(A/C)CATAAG(G/G)(C/C)G(T/C)(A/A)TTATGTTAATT
ODN2	AATTAACATAAGGCGTATTATGTTAATT
ODN3	ACTTCGTATAATCGCCATTATGTTAAAT
ODN4	ATTTCGCATAAGGCGTATTATGCGAAAT
ODN5	AATTAACATAAGGCGTATTATGTTAATT

Only the top strand is shown in the table. Nucleotides in parenthesis indicate mismatched positions. Phosphorothioate residues are underlined.

## Supplementary Materials

The following are available online at https://www.mdpi.com/2079-6382/9/7/405/s1, Figure S1: Phylogenetic trees of XerC_Ab_ and XerD_Ab_, Figure S2: Partial purification of XerC_Ab_ and XerD_Ab_.
