# Supplementary material for: Functional Analysis of the Acinetobacter baumannii XerC and XerD Site-Specific Recombinases: Potential Role in Dissemination of Resistance Genes
Source: Antibiotics (Basel). 2020 Jul 13;9(7):405. doi: 10.3390/antibiotics9070405 (PMC7399989; doi:10.3390/antibiotics9070405)
Supplement: Supplementary file 1 [file antibiotics-09-00405-s001.pdf]

SUPPLEMENTAL MATERIAL

Functional Analysis of the *Acinetobacter baumannii* XerC and XerD Site-Specific Recombinases: Potential Role in Dissemination of Resistance Genes

David L. Lin [1,2], German M. Traglia [3], Rachel Baker [2], David J. Sherratt [2], Maria Soledad Ramirez [1] and Marcelo E. Tolmasky [1,2,*]

1 Center for Applied Biotechnology Studies, Department of Biological Science, California State University Fullerton, Fullerton, CA 92831, USA; davlin0916@gmail.com (D.L.L.); msramirez@Fullerton.edu (M.S.R.); mtolmasky@fullerton.edu (M.E.T.)

2 Department of Biochemistry, University of Oxford, Oxford OX1 3QU, United Kingdom; davlin0916@gmail.com (D.L.L.); rachel.baker@bioch.ox.ac.uk (R.B.); david.sherratt@bioch.ox.ac.uk (D.J.S.); mtolmasky@fullerton.edu (M.E.T.)

3 Departamento de Desarrollo Biotecnológico, Instituto de Higiene, Facultad de Medicina, Universidad de la República (UDeLaR), 11600 Uruguay; gertra13b@gmail.com

* Correspondence: mtolmasky@fullerton.edu

Received: 12 June 2020; Accepted: 11 July 2020; Published: date

Keywords: site-specific recombination; carbapenemase; ESKAPE; *Acinetobacter*; plasmid; Xer; dif; pdif; Re27; gene transfer; gene dissemination; horizontal dissemination; horizontal transfer

---

Comparative analysis of $XerC_{Ab}$ or $XerD_{Ab}$ amino acid sequences

All $XerC_{Ab}$ and $XerD_{Ab}$ amino acid sequences available from the GenBank database were extracted and the redundant sequences were eliminated using CD-HIT software with a cutoff of one amino acid [1]. This process resulted in 161 $XerC_{Ab}$ and 70 $XerD_{Ab}$ unique amino acid sequences. One representative of each variant was used was used to generate the multiple alignment and determine identity and similarity using CLUSTALX [2]. The phylogenetic analysis was carried out using RaxML [3] and the phylogenetic tree was generated using iTOL [4]. The model of molecular evolution was done using Prottest3 [5]. The similarity values oscillated between 90% and 100% for both groups of proteins, which shows that there is very low variability among all variants. The phylogenetic trees generated by these analyses indicate that in both cases each group of sequences belongs to one phylogenetic group (Figure S1).

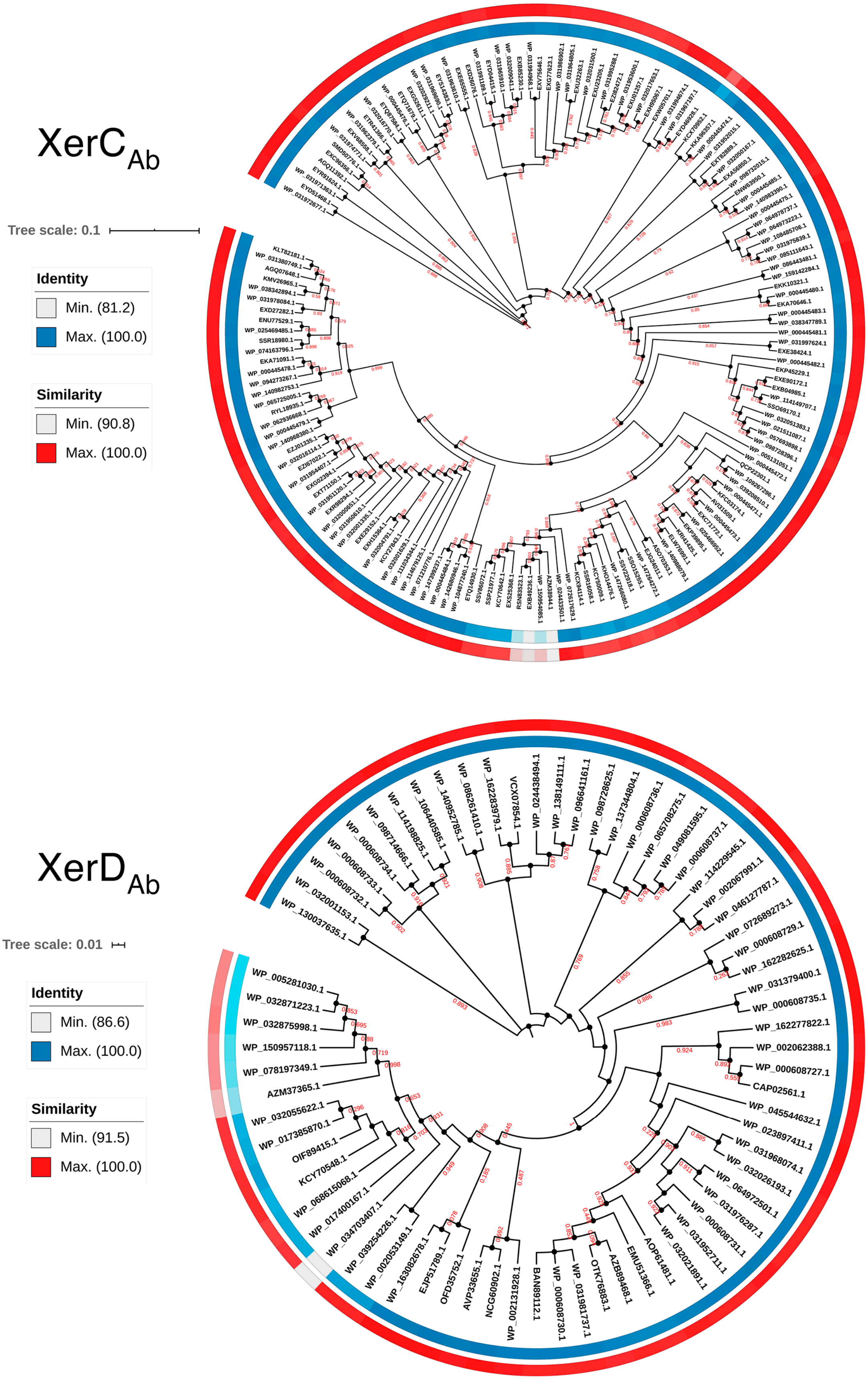

Figure S1. Phylogenetic trees of XerCAb and XerDAb. Amino acid sequence alignments were performed using ClustalX software from 161 (XerCAb) and 70 (XerDAb) amino acid sequences. The evolutionary history was inferred by Maximum Likelihood method using to RaxML software. The molecular

evolution model using to phylogenetic analysis was WAG (Whelan And Goldman) model that was predicted by Prottest3 software. The bootstrap method was used as support method (1000 runs).

Partial purification of XerC$_{Ab}$ or XerD$_{Ab}$

DNA fragments containing the *xerC*$_{Ab}$ or *xerD*$_{Ab}$ genes were generated by PCR amplification with the QIAGEN Taq master mix using as template genomic DNA from *A. baumannii* A118. Amplicons were inserted in pCR2.1 and then subcloned into the *Eco*RI site of pACYC184. Both *xerC*$_{Ab}$ or *xerD*$_{Ab}$ were further subcloned into pBAD102 with a C-terminal 6x histidine tag for overexpression and purification. The inserts of all recombinant plasmids were sequenced to ensure accuracy. Nucleotide sequencing was performed at the DNA Sequencing Facility, Department of Biochemistry, University of Oxford. The tagged XerC$_{Ab}$ and XerD$_{Ab}$ were purified by affinity using TALON metal affinity resin as previously described [6]. Briefly, *E. coli* DS9040 (pBAD102*xerC*$_{Ab}$) or *E. coli* DS9040 (pBAD102*xerD*$_{Ab}$) were cultured overnight at 37°C with shaking. Each culture was then diluted 1:100 and shaken at 200 rpm at 37°C for 3.5 hours. At this moment, protein expression was induced by addition of 0.1% arabinose and incubation at 30°C. After 4 hours, the cells were collected by centrifugation at 5,000 rpm for 20 minutes and resuspended in a buffer containing 50 mM Tris 7.5, 1 M NaCl, and 10% glycerol with protease inhibitor cocktail (Sigma). The cells were lysed using a French Press and the lysate was subjected to centrifugation at 19,000 rpm for 30 minutes at 4°C. The supernatant containing the protein of interest was mixed with TALON metal affinity resin and incubated for 1 hour. The resin was washed with a buffer containing 50 mM Tris-HCl pH 7.5 buffer, 500 mM NaCl, 10% glycerol, and 10 mM imidazole. The proteins were eluted by gravity column into 8 fractions with a buffer containing 50 mM Tris-HCl pH 7.5 buffer, 500 mM NaCl, 10% glycerol, and 200 mM imidazole. Proteins were analyzed using sodium dodecyl sulfate-15% polyacrylamide gel electrophoresis stained with coomassie blue to identify fractions containing XerC$_{Ab}$ and XerD$_{Ab}$ (Figure S2). The selected fractions were dialyzed with 10 mM Tris-HCl pH 7.5 using Zeba desalting columns (ThermoFisher Scientific), and concentrated to approximately 150 g/ml using Pierce protein concentrator PES columns (ThermoFisher Scientific) according to manufacturer recommendations.

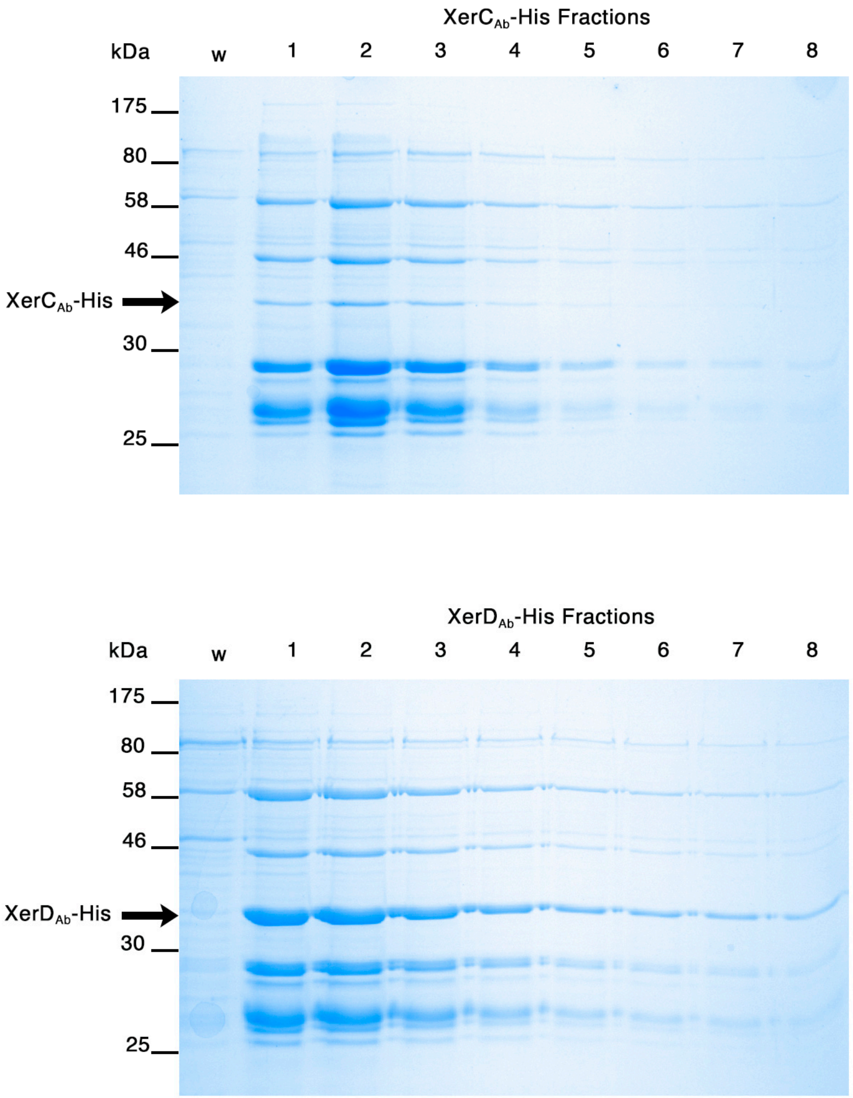

Figure S2. Partial purification of XerCAb and XerDAb. The top and bottom gels show aliquots of the fractions obtained after affinity chromatography. The proteins were fused to a histidine tag at the C-termini. Fractions 2 were selected for further processing.

References

1. Li, W.; Godzik, A. Cd-hit: a fast program for clustering and comparing large sets of protein or nucleotide sequences. *Bioinformatics* **2006**, *22*, 1658–1659.
2. Larkin, M.A.; Blackshields, G.; Brown, N.P.; Chenna, R.; McGettigan, P.A.; McWilliam, H.; Valentin, F.; Wallace, I.M.; Wilm, A.; Lopez, R., *et al.* Clustal W and Clustal X version 2.0. *Bioinformatics* **2007**, *23*, 2947–2948.
3. Stamatakis, A. RAxML-VI-HPC: maximum likelihood-based phylogenetic analyses with thousands of taxa and mixed models. *Bioinformatics* **2006**, *22*, 2688–2690.
4. Letunic, I.; Bork, P. Interactive Tree Of Life (iTOL) v4: recent updates and new developments. *Nucleic Acids Res.* **2019**, *47*, W256–W259.
5. Darriba, D.; Taboada, G.L.; Doallo, R.; Posada, D. ProtTest 3: fast selection of best-fit models of protein evolution. *Bioinformatics* **2011**, *27*, 1164–1165.
6. Arciszewska, L.K.; Baker, R.A.; Hallet, B.; Sherratt, D.J. Coordinated control of XerC and XerD catalytic activities during Holliday junction resolution. *J. Mol. Biol.* **2000**, *299*, 391–403.
